# A dataset for accounting, finance and economics research on US data breaches

**DOI:** 10.1016/j.dib.2021.106924

**Published:** 2021-03-01

**Authors:** Pierangelo Rosati, Theo Lynn

**Affiliations:** Irish Institute of Digital Business, Dublin City University, Ireland

**Keywords:** Cyber security, Data breaches, Finance, Accounting, Event study

## Abstract

This data article describes a dataset of data breaches in US listed firms over a ten-year period. Data breaches represent major events that pose serious challenges to organisations. The number of incidents has been on the increase over the last decade and this has attracted the interest of the media, consumers and regulators. While there is a well-established literature on cybersecurity in Computer Science and Information Systems journals, studies exploring the economic and business impacts of data breaches represent a relatively recent phenomenon. There is a nascent but fast-growing literature in accounting, finance and economics that focuses on the financial impacts of data breaches and this dataset provides a useful resource for future studies in this space. By providing data on the company identifier, the type of breach, the dates of breach disclosure, and relates these dates to the company's fiscal year, the dataset can be merged quickly with existing accounting and finance datasets. The dataset includes data on 506 incidents over a ten-year period thereby enabling cross-sectional and longitudinal analyses.

## Specifications Table

**Table:** 

Subject	Business, Management and decision sciences
Specific subject area	Accounting, Finance, Cyber Security
Type of data	CSV File (Table format)
How data were acquired	The original list of data breaches was retrieved from the Privacy Rights Clearinghouse repository (PRC).
Data format	The original list of data breaches provided by PRC was filtered to extract incidents that affected firms listed on the New York Stock Exchange (NYSE) or NASDAQ.
Parameters for data collection	The original list of incidents was compiled by PRC which collects information on cyber security incidents in US organisations through government agencies or verifiable media sources. The list of incidents was manually matched against the list of companies listed in the New York Stock Exchange (NYSE) or NASDAQ in the year of the incident.
Description of data collection	The original dataset included all 4552 cyber-security incidents disclosed by firms, non-profit organisations, healthcare organisations and government agencies in the US as published by PRC. The dataset was then filtered to only include events that affected companies listed on the New York Stock Exchange (NYSE) or NASDAQ between April 2005 and March 2015. The list was then enriched by (1) including standard company identifiers such as ticker symbol (a unique code assigned to a publicly-traded stock for trading purposes), gvkey (a unique six-digit number assigned to each company in the Compustat database) and CUSIP code (a unique identification number assigned to all traded securities in the United States), and (2) searching on LexisNexis^1^ whether the incident was made public before the announcement date and whether the affected company made any other announcement prior to the incident. More specifically we used LexisNexis Power Search to check whether any media article containing the company name and any of the following keywords in the title, headline or body of the article within 10 days prior to the announcement: M&A OR merger OR merged OR acquisition OR acquired OR CEO resignation OR CEO resigned OR CFO resigned OR delisting OR delisted OR IPO OR initial public offering OR earnings announcement OR earnings OR financial statement OR restatement OR restated OR investigation.
Data source location	The raw list of data breaches is available at: https://privacyrights.org/data-breaches. Ticker symbols were retrieved from the New York Stock Exchange (https://www.nyse.com/listings_directory/stock) and NASDAQ (https://www.nasdaq.com/market-activity/stocks/screener) websites. The gvkey and CUSIP identifiers were retrieved form the Compustat database (https://www.refinitiv.com/en/financial-data/company-data/fundamentals-data/standardized-fundamentals/sp-compustat-database). Media articles mentioning the company name were retrieved from LexisNexis (https://www.lexisnexis.com/en-us/professional/nexis/nexis.page). The final dataset presented in this article is available on Mendeley at the following link: https://data.mendeley.com/datasets/w33nhh3282/1.
Data accessibility	Repository name: Mendeley Data Direct URL to data: http://dx.doi.org/10.17632/w33nhh3282.1.

1 https://www.lexisnexis.com/en-us/professional/nexis/nexis.page.

## Value of the Data

• The dataset augments the Privacy Rights Clearinghouse list of data breaches relating to US public companies with commonly used identifiers for those companies, verified dates of official announcements of the data breaches, and additional data relating the data breach disclosures with key dates in the affected firm's fiscal year. The use of common identifiers allows this dataset to be merged with other datasets using the same identifiers thus accelerating time to analysis.
• The dataset serves as a reference for researchers looking to examine the impact of data breaches on the performance of US listed companies and encourages exploration on the relationship between the timing of disclosures, corporate decision making, corporate reporting, and market activity. The dataset includes 506 incidents over a ten-year period and therefore is suitable both for cross-sectional and longitudinal analyses.
• This dataset can be used by research institutions, regulators, financial analysts, and journalists for research on the relationship and impact of data breaches on US publicly listed firms from multiple perspectives.

## 1 Data Description

The dataset presented in this article consists of 506 data breaches and associated characteristics that affected US listed companies over a 10-year period from April 2005 to March 2015 [8]. Table 1 provides an overview of the number of incidents per year while Table 2 provides an overview of the number of incidents by type.

**Table 1:** Number of incidents by year.

Year	No. of Incidents
2005	28
2006	76
2007	66
2008	36
2009	20
2010	65
2011	60
2012	48
2013	61
2014	43
2015	3
**Total**	**506**

**Table 2:** Number of incidents by type.

Breach Type	No. of Incidents
CARD	25
DISC	80
HACK	118
INSD	83
PHYS	30
PORT	139
STAT	16
UNKN	15
**Total**	**506**

## 2 Experimental Design, Materials and Methods

The dataset was gathered from the Privacy Rights Clearinghouse (PRC) and then augmented with manual data collection. PRC is a non-profit organisation based in California that aims to identify trends in privacy protection to support advocates, policymakers, industry, media and consumers. PRC have also compiled an historical archive of cyber security incidents affecting US organisations which is available on their website. Information about each incident is collected through either government agencies or verifiable media sources. This archive cannot be considered exhaustive as “many organisations are not aware they have been breached or are not required to report it based on reporting laws” [9] but it still represent a valuable asset for academic researchers. The value of the PRC repository^1^ for academic research has been demonstrated by a number of published articles already (see, for example, [1], [2], [3], [4], [5], [6], [7]).

The original dataset contained 4552 cyber-security incidents disclosed by firms, non-profit organisations, healthcare organisations and government agencies in the US and it is particularly suitable for event studies, a common research methodology in the accounting and finance domains. However, researchers seeking to work with the original dataset may encounter four main challenges: (1) the lack of an identifier for listed companies; (2) the lack of a common company identifier to merge the PRC list with widely-used databases such as Compustat^2^; (3) the need to verify the date of the initial announcement as some of the events may have been mentioned by the media before the official disclosure; and (4) some of the affected organisations may have made other announcements in the days prior the breach announcement and this may ultimately alter stock price reaction to the disclosure.

In order to overcome these challenges, we manually filtered companies listed in New York Stock Exchange (NYSE)^3^ and NASDAQ Stock Exchange.^4^ We then enriched our dataset by manually collecting identifiers that are commonly used in different accounting and finance databases, namely: (1) stock ticker symbol which is a unique code assigned to a publicly-traded stock for trading purposes; (2) Global Company Key (or gvkey) which is a unique six-digit number assigned to each company in the Compustat database; and (3) CUSIP (Committee on Uniform Securities Identification Procedures) number which is a unique identification number assigned to all traded securities in the United States. We also identified additional information such as the fiscal year in which each incident occurred (which may be different from a calendar year), and verified whether the reported announcement date corresponds to the date of the first article or official announcement, and if the affected company had made any other announcement in the 10 days prior to the breach announcement. This process ultimately increases the quality and reliability of the dataset while also allowing researchers to merge the events with financial information gathered from databases like Compustat, CRSP,^5^ Datastream^6^ etc.

The dataset consists of a single table containing the following fields for every data breach (where information is available):

• Event_ID: Event identifier;
• ticker: Ticker symbol of the affected company.
• gvkey: Gvkey of the affected company as retrieved from Compustat.
• fiscal_month: Month of year corresponding to fiscal-year end.
• cusip: CUSIP code of the affected company.
• name: Name of the affected company.
• fiscal_year: Fiscal year in which the incident occurred.
• event_year: Calendar year in which the incident occurred.
• event_date: Date in which the incident occurred.
• confound_dum: This field is equal to 1 if the affected company released any major announcement in the 10 days prior to the breach.^7^
• confound_type: This field contains the type of announcement released in the 10 days prior to the breach (if any):
  ○ Earnings: Earnings announcement;
  ○ Investigation: Regulatory investigation;
  ○ IPO: Initial Public Offering;
  ○ M&A: Merger or acquisition announcement;
  ○ Restatement: Restatement of previously issued financial statement(s);
  ○ Statement: Release of quarterly or annual financial results;
  ○ Other: Other major announcement not included in the categories above.
• breach_size: Number of records affected by the breach (if available).
• breach_type: Type of data breach as classified by the Privacy Rights Clearinghouse:
  ○ CARD: Fraud Involving credit/debit cards not via hacking.
  ○ HACK: Hacked by a malicious outside party or infected by a malware.
  ○ INSD: Incident due to a malicious insider.
  ○ PHYS: Incident due to lost, discarded or stolen physical device or documents.
  ○ PORT: Incident due to lost, discarded or stolen laptop, smartphone, memory stick, CDs, hard drive etc.
  ○ STAT: Incident due to lost, inappropriately accessed, discarded or stolen computer or server not designed for mobility.
  ○ DISC: Unintended disclosure of sensitive information not Involving hacking, intentional breach or physical loss.
  ○ UNKN: Unknown cause.
• event_state: The US state in which the breach occurred.
• hq_state: The state in which the headquarters of the affected company is located.

## Ethics Statement

The dataset only includes organisational information that has been disclosed to the public. As such, there is no ethical concerns associated with the dataset.

## Declaration of Competing Interest

The authors declare that they have no known competing financial interests or personal relationships which have, or could be perceived to have, influenced the work reported in this article.

## Footnotes

1 https://privacyrights.org/data-breaches.

2 https://www.refinitiv.com/en/financial-data/company-data/fundamentals-data/standardized-fundamentals/sp-compustat-database.

3 https://www.nyse.com/listings_directory/stock.

4 https://www.nasdaq.com/market-activity/stocks/screener.

5 http://crsp.org/.

6 https://www.refinitiv.com/en/products/datastream-macroeconomic-analysis.

## Footnotes

7 The following keyword search was implemented in Lexis Nexis Power Search to check the title, headline or body of articles published by English-language media: *Company_name* AND M&A OR merger OR merged OR acquisition OR acquired OR CEO resignation OR CEO resigned OR CFO resigned OR delisting OR delisted OR IPO OR initial public offering OR earnings announcement OR earnings OR financial statement OR restatement OR restated OR investigation.
