# Corrigendum to “A dataset for accounting, finance and economics research on US data breaches” [Data in Brief 35 (2021) 1–6/ 106924]

**DOI:** 10.1016/j.dib.2021.107688

**Published:** 2021-12-07

**Authors:** Pierangelo Rosati, Theo Lynn

**Affiliations:** a Irish Institute of Digital Business, Dublin City University, Ireland; b Irish Institute of Digital Business, Dublin City University, Ireland

The authors regret that there was an error in the description of data collection process in Rosati and Lynn [1]. This Corrigendum provides clarity with regard to the amendments that need to be considered.

1. In the Experimental Design, Materials and Methods section, the sentence “[…] and if the affected company had made any other announcement in the 10 days prior to the breach announcement” should read as follows “[…] and if the affected company had made any other announcement in the 7 days prior to the breach announcement”.
2. In the Experimental Design, Materials and Methods section, the sentence “confound_dum: This field is equal to 1 if the affected company released any major announcement in the 10 days prior to the breach” should read as follows “confound_dum: This field is equal to 1 if the affected company released any major announcement in the 7 days prior to the breach”.
3. In the Experimental Design, Materials and Methods section, the sentence “confound_type: This field contains the type of announcement released in the 10 days prior to the breach (if any)” should read as follows: “confound_type: This field contains the type of announcement released in the 7 days prior to the breach (if any)”.

The authors would like to apologise for any inconvenience caused.
